# Cerebral border zone infarction: an etiological study

**DOI:** 10.1186/s41983-018-0008-0

**Published:** 2018-04-25

**Authors:** Tarek Mohammed El-Gammal, Wafik Said Bahnasy, Osama Abd Allah Ragab, Ayman Mohammed AL-Malt

**Affiliations:** Department of Neuropsychiatry, Faculty of Medicine, Tanta University, Tanta, 31527 Egypt

**Keywords:** Border zone infarction, Brain CT angiography, Transcranial duplex and extracranial duplex

## Abstract

**Background:**

Border zone infarcts (BZI) are ischemic lesions at the junction between two main arterial territories which may be either cortical or internal BZI.

**Methods:**

This study was conducted on 76 cerebral BZI patients and 20 healthy control subjects. Patients were divided to group I included 26 internal BZI, group II included 19 cortical BZI and group III included 21 mixed internal/cortical BZI patients. Included subjects were submitted to neurological examination, laboratory investigations, ECG, echocardiogram, brain CT and/or MRI and extra and intracranial blood vessels imaging by duplex and CT angiography.

**Results:**

Hypertension was significantly higher among groups I and III compared to group II while atrial fibrillation (AF) was significantly higher in groups II and III than group I (*p* < 0.05). Sonographic duplex assessment of extra and intracranial blood vessels revealed significant increase in mean flow velocities of CCA, ICC and MCA on both side in groups I and III compared to group II (*p* < 0.05). CT angiography revealed non-significant differences between BZI patients and control as well as in between the three BZI patient’s groups regarding the existence of vertebral artery hypoplasia and/or circle of Willis anomalies.

**Conclusions:**

Vascular stenosis is the main etiological factor in internal BZI while AF is the predominant etiological factor of cortical BZI. Congenital vascular anomalies play roles in the localization of BZI but cannot predispose to it except when comorbid with hemodynamic disturbances.

## Background

Border-zone infarctions (BZI) are ischemic lesions located at the junction between two arterial territories and represent about 10% of all brain infarcts (Cauquil-Michon et al. 2011). Border-zone infarctions are either internal or external (cortical) BZI. The former appears as multiple infarcts in a rosary like pattern in the centrum semiovale and corona radiata while the latter are usually wedge shaped, located in the cerebral cortex between the territories of the three main cerebral arteries (Mangla et al. 2011).

The etiology of BZI is still under researching but some studies supposed internal BZI to be due to hemodynamic compromise secondary to proximal stenosis and/or hypo-perfusion due to low cardiac output, whereas cortical BZI were linked to embolization either from the heart or atherosclerotic plaques in large arteries (Hossmann and Heiss 2016).

Brain collateral circulation plays an important role in guarding against BZI development by maintaining adequate cerebral perfusion with flow redistribution to ischemic areas. The Circle of Willis (COW) is a major site of collateral flow but its anomalies are common affecting its ability to maintain sufficient perfusion and increasing the risk of BZI (Wang and Wang 2015).

### Aim of the work

Was to investigate some of the etiological causes of BZI (different types) focusing on the role of developmental and/or acquired disorders of arterial vessels supplying the brain.

## Subjects and methods

This work was a prospective study conducted on 76 consecutives cerebral BZI patients attending the neurovascular unit and intensive care units of the Neurology Department and The Center of Neurology and Psychiatry, Tanta University Hospitals, in the period from 1st June to the end of December 2016. Patients were divided to three groups: 26 internal BZI (group I), 19 cortical BZI (group II), and 21 mixed internal/cortical BZI (group III). Twenty healthy control subjects matching the patient’s age and sex were also included.

The study protocol was approved by The Research Ethics Committee and Quality Assurance Unit, Faculty of Medicine, Tanta University. Participation was voluntary and all contributors or their first-degree relatives received detailed information about the aims of this research work, and an informed consent was obtained prior to the commencement of the study.

All patients were subjected to history taking, thorough medical and neurological examinations, ECG, echocardiography, brain CT and/or MRI and routine laboratory investigations including CBC, renal functions, liver functions, lipid profile, blood sugar, and HBA1c.

Extracranial vessels duplex study was evaluated by multifrequency 3–12 MHz, real-time linear array ultrasound transducer with sagittal, coronal, and axial views while intracranial vessels duplex imaging was assessed by multifrequency 1–3 MHz phased array transcranial transducer with trans-axial mesencephalic view through temporal window.

Patients were subjected to CT angiography for extra and intracranial blood vessels for assessment of carotid and vertebral arteries as well as the integrity of circle of Willis using GE Medical System (Waukesha, WI, USA). Images were obtained from C3 to the vertex using the following scanning parameters: detector rows, 16 collimations, 0.625-mm pitch, 0.93 gentry rotation time, 1.0 s slice thickness, 0.625 mm tube load 380 mA, and tube voltage 120 kV. A total volume of 80–100 mL of non-ionic contrast medium was injected at a rate of 4.0–4.5 mL/s through an antecubital vein, with 18-s scan delays or the use of Smart Prep at the pulmonary artery. Coronal and sagittal multiplanar reformatted (MPR), maximum intensity projections (MIP), and 3D volume rendered images were created at GE advantage workstation.

Statistical analysis was conducted using SPSS version 19 (Statistical Package for Social Studies) created by IBM, Illinois, Chicago, USA. For numerical values the range mean, and standard deviations were calculated. For categorical variable, the number and percentage were calculated and differences between subcategories were tested using the *z*-score test, ANOVA and Tukey’s tests. *P* value < 0.05 was considered statistically significant.

## Results

The study included 76 BZI patients divided to three groups: 26 internal BZI (group I), 19 cortical BZI (group II), and 21 mixed internal/cortical BZI (group III). The three studied groups showed non-significant difference regarding patients’ age and sex with *p* value 0.124 and 0.276 respectively (Table 1).

**Table 1:** Characters and data of the studied internal (group I), cortical (group II), and mixed internal/cortical (group III) border zone infarction patients

		Group I (*n* = 26)	Group II (*n* = 19)	Group III (*n* = 21)	*X*2	*p* value
Age		61.69 ± 6.1	58.79 ± 5.6	62.48 ± 5.9	2.15	0.124
Sex	Male	16 (64.5%)	10 (52.3%)	8 (38.1%)	2.56	0.276
	Female	10 (38.5%)	9 (47.4%)	13 (61.9%)		
Hypertension		18 (69.2%)	8 (42.1%)	16 (76.2%)	6.51	0.038*
Diabetes		8 (30.8%)	6 (31.6%)	7 (33.3%)	0.036	0.982
Dyslipidemia		13 (5%)	7 (36.8%)	14 (66.7%)	3.59	0.166
Smoking		8 (30.8%)	5 (26.3%)	6 (28.6%)	0.107	0.947
IHD		5 (19.2%)	6 (31.6%)	7 (33.3%)	1.414	0.493
Atrial fibrillation		7 (26.3%)	12 (63.2%)	13 (61.9%)	6.05	0.048*
Previous CVA		3 (11.5%)	3 (15.8%)	2 (9.5%)	0.381	0.826
Cerebral infarct	Right	11 (42.3%)	5 (26.3%)	3 (14.3%)	7.37	0.117
	Left	8 (30.8%)	7 (36.8%)	5 (23.8%)		
	Bilateral	7 (26.9%)	7 (36.8%)	13 (61.9%)		
Anterior circulation BZI		7 (26.9%)	5 (26.3%)	2 (9.5%)	3.94	0.413
Posterior circulation BZI		7 (26.9%)	5 (26.3%)	4 (19.04%)		
Anterior-posterior junctions BZI		12 (46.2%)	9 (47.4%)	15 (71.4%)		

*BZI* border zone infarction, *CVA* cerebrovascular accident, *IHD* ischemic heart disease

*Significant

The study showed that history of hypertension especially poorly controlled ones was significantly higher among groups I and III patients than group II (69.23, 76.19, and 42.11% respectively) with *p* value 0.038. On the other hand, the presence of atrial fibrillation (AF) was significantly higher in groups II and III than group I (63.15, 61.9 and 26.29% respectively) with *p* value 0.048 (Table 1).

The present study revealed significant increase in the incidence of each of diabetes mellitus (DM), dyslipidemia, ischemic heart diseases, and previous cerebrovascular accidents in BZI patients compared to healthy control subjects (*p* values were < 0.001, < 0.001, 0.0032 and < 0.001 respectively). At the same time, there were non-observable differences between the three BZI patients’ groups with *p* > 0.05 as shown in Table 1. The study also showed non-significant differences between the three BZI patients’ groups regarding sites and laterality of the infarction (Table 1).

Ultrasonography revealed non-significant difference between the 3 studied patients’ groups regarding the carotid intima media thickness (*p* value > 0.05). Patients with internal BZI and mixed internal/cortical BZI showed significant increase in the mean flow velocity (MFV) of each of common carotid arteries (CCA), internal carotid arteries (ICA), and middle cerebral arteries (MCA) compared with cortical BZI patients with *p* values < 0.05. The MFV of vertebral arteries (VA), basilar arteries, anterior cerebral arteries (ACA), and posterior cerebral arteries (PCA) did not show significant differences between the three studied BZI patients’ groups (Table 2). Increased MFV of the affected arteries were higher among studied hypertensive patients especially poorly controlled ones.

**Table 2:** Extra and intracranial duplex findings among studied internal (group I), cortical (group II), and mixed internal/cortical (group III) border zone infarction patients

		Group I (*n* = 26)	Group II (*n* = 19)	Group III (*n* = 21)	*f* value	*p* value
CCA-IMT	Right	0.09 ± 0.02	0.09 ± 0.01	0.07 ± 0.04	2.06	0.134
	Left	0.1 ± 0.01	0.09 ± 0.01	0.13 ± 0.17	1.04	0.359
CCA-MFV	Right	48.59 ± 6.47	41.06 ± 5.24	49.85 ± 5.57	9.21	0.0003*
	Left	48.29 ± 2.92	39.71 ± 7.39	48.77 ± 2.75	26.79	< 0.0001*
ICA-MFV	Right	57.84 ± 13.91	40.58 ± 14.48	52.5 ± 17.1	6.98	0.0018*
	Left	59.38 ± 19.97	45.98 ± 12.29	54.65 ± 16.47	3.611	0.032*
VA-MFV	Right	25.32 ± 4.08	25.37 ± 5.09	24.92 ± 4.05	0.051	0.949
	Left	27.75 ± 8.68	26.33 ± 5.63	27.55 ± 8.57	0.19	0.826
Basilar artery MFV		23.12 ± 6.91	23.8 ± 21.97	23.4 ± 7.48	0.061	0.940
MCA-MFV	Right	60.53 ± 23.13	47.98 ± 21.97	58.56 ± 19.67	5.43	0.0066*
	Left	59.92 ± 28.49	44.78 ± 14.69	57.08 ± 18.51	8.111	0.0007*
ACA-MFV	Right	40.67 ± 2.78	39.06 ± 4.14	40.62 ± 3	1.57	0.215
	Left	39.43 ± 3.32	38.19 ± 3.74	39.17 ± 3.08	0.77	0.466
PCA-MFV	Right	35.14 ± 4.1	33.03 ± 5.12	35.01 ± 4.27	1.43	0.246
	Left	35.25 ± 4.26	32.9 ± 4.14	35.13 ± 4.35	1.97	0.147

*ACA* anterior cerebral artery, *BA* basilar artery, *CCA* common carotid artery, *ICA* internal carotid artery, *IMA* intima media thickness, *MCA* middle cerebral artery, *MFV* mean flow velocity, *PCA* posterior cerebral artery, *VA* vertebral artery

*Significant

**Fig. 1:**
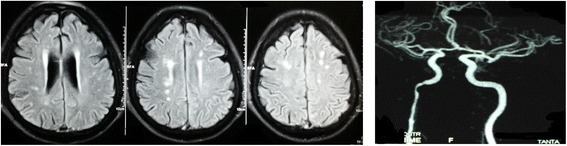
A case of bilateral internal border zone infarction with CT angiography showing severe stenosis in the cavernous and petrous portion of right internal carotid artery

Extra and intracranial arterial assessment by CT angiography showed significant increase in the number of patients having > 50% stenosis in each of ICA and/or MCA in groups II and III than group I (Fig. 1). Vertebral artery and basilar artery stenosis showed non-significant differences between the 3 studied patients’ groups (Table 3).

**Table 3:** CT angiography among studied internal (group I), cortical (group II), and mixed internal/cortical (group III) border zone infarction patients

		Group I (*n* = 26)	Group II (*n* = 19)	Group III (*n* = 21)	*f* value	*p* value
ICA stenosis	Right	10 (38.5%)	2 (10.5%)	9 (42.9%)	6.48	0.039*
	Left	9 (34.6%)	1 (5.3%)	7 (33.3%)	6.31	0.042*
MCA stenosis	Right	12 (42.3%)	2 (10.5%)	7 (33.3%)	6.52	0.038*
	Left	10 (38.5%)	1 (5.3%)	11 (52.4%)	174.9	0.0001*
VA stenosis	Right	2 (7.7%)	0	2 (9.5%)	1.79	0.408
	Left	1 (3.8%)	0	1 (4.8%)	0.867	0.648
Basilar artery stenosis		2 (7.7%)	0	2 (9.5%)	1.79	0.408
Vertebral artery hypoplasia		6 (23.1%)	3 (15.8%)	3 (19%)	1.846	0.764
Hypoplastic anterior communicating		6 (23.1%)	2 (10.53%)	6 (28.6%)	2.34	0.885
Hypoplastic posterior communicating		3 (11.5%)	3 (15.8%)	5 (23.8%)		

*ICA* internal carotid artery, *MCA* middle cerebral artery, *VA* vertebral artery

*Significant

The study also revealed non-significant difference between BZI patients and control regarding the presence of VA hypoplasia and/or hypoplastic circle of Willis (COW) segment (hypoplastic anterior communicating or posterior communicating arteries) with *p* value > 0.05 (Table 4). At the same time, there were non-observable difference between the three BZI patients’ groups regarding the existence of VA hypoplasia (23, 15.8, and 19% for groups I, II, and III respectively with *p* value 0.764) and/or hypoplastic COW segment (34.6, 26.3, and 52.3% in groups I, II, and III respectively with *p* value 0.885).

**Table 4:** CT angiography among the border zone infarction patients and control

	Patients (*n* = 76)	Control (*n* = 20)	*z* value	*p* value
Vertebral artery hypoplasia	13 (17.1%)	3 (15%)	0.2248	0.82588
Hypoplastic anterior communicating	8 (10.5%)	2 (10%)	0.0686	0.9442
Hypoplastic posterior communicating	11 (14.5%)	3 (15%)	0.0593	0.95216

## Discussion

Border-zone infarcts were traditionally attributed to reduced blood flow caused by severe stenosis or occlusion of proximal cranio-cervical arteries and/or hypo-perfusion under the circumstance of severe systemic hypotension. Micro-emboli, also from the heart or proximal stenotic arteries, has been postulated to be another etiological factor of BZI (Sorgun et al. 2015a).

**Fig. 2:**
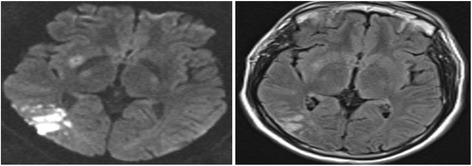
Brain MRI showing right parieto-occipital cortical border zone infarction

The study showed non-significant differences of gender or age between the different types of BZI (internal, cortical and mixed internal/cortical) (Fig. 2). These results are in accordance with that of Siegler et al. (2013) and Yong et al. (2006) who estimated that neither the age nor the sex of the patients was a risk factor of any type of BZI despite of being well-known non-modifiable risk factors of stroke in general.

The study also revealed that hypertension especially when poorly controlled was higher among internal and mixed internal/cortical BZI than pure cortical BZI patients. The former patients showed signs of CCA, ICA, and MCA stenosis as evidenced by increased duplex MFV and CT angiography. These results are in accordance with that of Franklin et al. (1997) and Catena et al. (2015) who concluded that hypertension is a strong risk factor of atherosclerosis and carotid artery stenosis which in turn are risks of internal BZI.

Atrial fibrillation was present in the three included BZI patients’ groups with higher incidence among those with cortical BZI (Fig. 2). Sorgun et al. (2015b) and Bergui et al. (2015) are in harmony with this result and reported a high vulnerability of cortical BZI among AF patient’s secondary to micro emboli. On the other side, Bladin and Chambers (1993) found high incidences of cardiac diseases in internal BZI patients, but this difference may be due to combined ischemic heart diseases and dysrhythmias in their included patients.

This study showed significant increase in the incidence of each of DM, dyslipidemia, ischemic heart diseases and previous cerebrovascular accidents in BZI patients compared to healthy control subjects. At the same time, there were non-significant differences between the three studied BZI patients’ groups regarding these stroke risk factors. These results are agreed by Sorgun et al. (2015b) and Weill et al. (2017) who reported non-significant differences between internal and cortical BZI regarding the incidence of each of DM, dyslipidemia, coronary heart diseases, and previous strokes or transient ischemic attacks.

Extra- and intra-cranial vessel examination using duplex studies and CT angiography showed high incidence of > 50% stenosis in the CCA, ICA, and MCA among internal and mixed internal/cortical BZI than cortical BZI patients. Förster et al. (2008) agreed with these results and estimated a positive correlation between the degree of ICA stenosis and the incidence of internal BZI as investigated by magnetic resonance angiography and Doppler ultrasound. Persoon et al. (2014) and Müller et al. (2015) explained this positive correlation by hypo-perfusion and impaired emboli clearance in the border zone areas. On the other side, Joinlambert et al. (2012) reported significant carotid stenosis in a high proportion of their studied cortical BZI patients possibly due to non-inclusion of internal BZI patients and the degree of stenosis in a high proportion of their studied patients was < 50%. Wang and Wang (2015) and Kim et al. (2010) concluded that ICA stenosis should be associated with MCA stenosis to be considered as a risk of internal BZI and this observation is somewhat in harmony with the results of the present study. Lopez et al. (2014) explained internal BZI by the extension of intracranial atherosclerosis to the origin of adjacent perforators but hypo-perfusion as a sole mechanism of BZI is relatively uncommon.

The present study showed non-significant increase in the existence of congenital arterial anomalies (VA hypoplasia or hypoplastic COW segment) in BZI patients when compared with healthy control. At the same time, there was no observable difference between internal and cortical BZI patients regarding the existence of congenital cerebral arterial anomalies. These results were in accordance with the work of Szabo et al. (2001) who found nearly normal incidence of COW anomalies in control subjects and they stated that there is no statistical correlation between the presence of hypoplastic vertebral artery or hypoplastic COW segment and the development of BZI. In controversy to the results of this study, Hartkamp et al. (1999) found an increased collateral flow in the anterior COW and increased vessel diameters in healthy control than BZI patients focusing to their adaptive and protective effect as a compensatory mechanism. Kumral et al. (2004) also found higher incidences of hypoplastic posterior communicating and non-detected anterior communicating arteries (45 and 50% respectively) in their studied BZI patients possibly due to dividing cortical BZI to anterior and posterior subgroups.

## Conclusions

The findings of the current study revealed high frequency of extracranial and intracranial vascular stenosis with internal BZI which is considered as the major etiological factor for this type of BZI. On the other hand, AF is more prevalent among cortical BZI pointing to embolism of the cerebral arteries as an expected cause. Finally, congenital vascular anomalies in the COW and/or vertebral artery hypoplasia mainly play roles in the localization of BZI but cannot predispose to its occurrence except when comorbid with hemodynamic disturbances.

## Abbreviations

ACA: Anterior cerebral artery
AF: Atrial fibrillation
BZI: Border zone infarction
CCA: Common carotid artery
COW: Circle of Willis
DM: Diabetes mellitus
ICA: Internal carotid artery
MCA: Middle cerebral artery
MFV: Mean flow velocity
PCA: Posterior cerebral artery
VA: Vertebral artery
